# The Clinical Significance of the Insulin-Like Growth Factor-1 Receptor Polymorphism in Non-Small-Cell Lung Cancer with Epidermal Growth Factor Receptor Mutation

**DOI:** 10.3390/ijms17050763

**Published:** 2016-05-18

**Authors:** Tu-Chen Liu, Ming-Ju Hsieh, Ming-Che Liu, Whei-Ling Chiang, Thomas Chang-Yao Tsao, Shun-Fa Yang

**Affiliations:** 1 Institute of Medicine, Chung Shan Medical University, Taichung 402, Taiwan; liou.dj@msa.hinet.net (T.-C.L.); 170780@cch.org.tw (M.-J.H.); 2 Department of Chest Medicine, Cheng-Ching General Hospital, Taichung 402, Taiwan; 3 Cancer Research Center, Changhua Christian Hospital, Changhua 500, Taiwan; 4 Department of Biochemistry and Molecular Biology, University of Massachusetts, Amherst, MA 01003, USA; oldyei@gmail.com; 5 School of Medical Laboratory and Biotechnology, Chung Shan Medical University, Taichung 402, Taiwan; wlchiang@csmu.edu.tw; 6 School of Medicine, Chung Shan Medical University, Taichung 402, Taiwan; 7 Division of Chest, Department of Internal Medicine, Chung Shan Medical University Hospital, Taichung 402, Taiwan; 8 Department of Medical Research, Chung Shan Medical University Hospital, Taichung 402, Taiwan

**Keywords:** non-small-cell lung carcinoma, insulin-like growth factor 1 receptor, epidermal growth factor receptor, genetic variants

## Abstract

The insulin-like growth factor 1 (IGF1) signaling pathway mediates multiple cancer cell biological processes. IGF1 receptor (IGF1R) expression has been used as a reporter of the clinical significance of non-small-cell lung carcinoma (NSCLC). However, the association between *IGF1R* genetic variants and the clinical utility of NSCLC positive for epidermal growth factor receptor (EGFR) mutation is not clear. The current study investigated the association between the *IGF1R* genetic variants, the occurrence of EGFR mutations, and clinicopathological characteristics in NSCLC patients. A total of 452 participants, including 362 adenocarcinoma lung cancer and 90 squamous cell carcinoma lung cancer patients, were selected for analysis of *IGF1R* genetic variants (rs7166348, rs2229765, and rs8038415) using real-time polymerase chain reaction (PCR)genotyping. The results indicated that GA + AA genotypes of *IGF1R* rs2229765 were significantly associated with EGFR mutation in female lung adenocarcinoma patients (odds ratio (OR) = 0.39, 95% confidence interval (CI) = 0.17–0.87). Moreover, The GA + AA genotype *IGF1R* rs2229765 was significantly associated with EGFR L858R mutation (*p* = 0.02) but not with the exon 19 in-frame deletion. Furthermore, among patients without EGFR mutation, those who have at least one polymorphic A allele of *IGF1R* rs7166348 have an increased incidence of lymph node metastasis when compared with those patients homozygous for GG (OR, 2.75; 95% CI, 1.20–2.31). Our results showed that *IGF1R* genetic variants are related to EGFR mutation in female lung adenocarcinoma patients and may be a predictive factor for tumor lymph node metastasis in Taiwanese patients with NSCLC.

## 1. Introduction

Lung cancer is a common type of cancer worldwide [1]. Two primary types of lung cancer are small cell lung carcinoma (SCLC) and non-small-cell lung carcinoma (NSCLC). Among lung cancers, NSCLC accounts for approximately 80% [2]. Common types of NSCLC include adenocarcinoma, squamous cell carcinoma, and large cell carcinoma.

The epidermal growth factor receptor (EGFR) is a transmembrane growth factor receptor with tyrosine kinase (TK) activity that plays a critical role for transmitting growth factor signaling from the extracellular region into the cell [3]. In the intracellular region, the following signaling transduction is regulated mainly through the PI3K-AKT-mTOR pathway, the RAS-RAF-MEK-ERK pathway and the signal transducer and activator of transcription (STAT) pathway [4]. The EGFR singling system is important for cell proliferation, differentiation, and anti-apoptosis [4,5]. It has been reported that mutation in the TK domain of *EGFR* gene is associated with NSCLC [6,7,8]. The most common mutations in the TK domain of EGFR are the in-frame deletion mutation in exon 19 and the substitution mutation (L858R) in exon 21. Mutation in the TK domain of EGFR causes the conformational change in protein structure. This results in constitutive TK activity and its downstream signaling pathway [9,10]. The EGFR is considered a therapeutic target for treatment in NSCLC. It has been reported that gefitinib and erlotinib are EGFR tyrosine kinase inhibitors (TKIs) for in-frame deletion in exon 19 and the substitution mutation (L858R) in exon 21 [6,7,8].

The insulin-like growth factor 1 (IGF1) signaling pathway mediates multiple cell biological processes including proliferation, differentiation, and metabolism [11]. The IGF1 system is composed of ligands, receptors, and a family of IGF binding proteins (IFGBPs). While IFG1 binds to insulin-like growth factor 1 receptor (IGF1R) on the cell membrane, the receptor-type tyrosine kinase IGF1R will be activated by autophosphorylation and switch on the downstream intracellular signaling transduction pathways, including the PI3K-PDK1-AKT pathway and the RAS-RAF-MEK-ERK pathway [12,13]. These pathways are vital for cell proliferation, differentiation, and anti-apoptosis [11,14]. Numerous studies have demonstrated that dysfunction of the IGF1 signaling pathway results in various diseases, including cancer, metabolic disease, as well as neurodegenerative diseases [15,16,17]. In addition, IGF1 system dysregulation has been reported in cancers such as NSCLC and in other tumors [18]. Recently, the clinical significance of IFG1R expression in human NSCLC has been reported [19]. The results showed that high membranous IGF1R expression was predictive of poor progression-free survival (PFS) in adenocarcinoma, but had better PFS in squamous cell carcinoma [19]. Moreover, Reinmuth *et al.* [20] also characterized the IGF1R mutations, single nucleotide polymorphisms (SNPs), and protein expression in resected NSCLC and found that patients with adenocarcinomas and homozygous for the rs8038415 T-allele had significantly better survival, but found no different in disease free survival. These findings indicate that IGF1R could be a potential therapeutic target and will guide further investigation.

Contributions of the IGF1R expression to the formation of NSCLC have been well established [18]. However, the correlation between *IGF1R* gene polymorphisms and the hotspot mutations of EGFR (in-frame deletion mutation in exon 19 and L858R mutation) of NSCLC have not been clarified. In the present study, the selection of two common polymorphisms (rs7166348 and rs8038415) from the *IGF1R* gene is based on their wide associations with the development of cancer [20,21]. Moreover, synonymous with the SNP, rs2229765 (E598E in exon 16) was selected in this study since it was found to be associated with levels of free IGF-1 [22]. Thus, in this study we aimed to explore the association between the genetic SNPs of *IGF1R* (rs7166348, rs2229765, and rs8038415) and the TK-domain mutations of EGFR in NSCLC. These results may provide a clue to understanding the potential consequences of lung cancer.

## 2. Results

A total of 452 patients were enrolled in this study. The demographics and clinical characteristics of patients were shown in Table 1. The average age of patients was 66 years. The gender distribution in patients was 251 male (55.5%) and 201 female (44.5%) with a sex ratio of about 1. Across all patients, the percentages of adenocarcinoma and squamous cell carcinoma were 81.1% (362/452) and 19.9% (90/452) respectively. Moreover, female patients possessed higher frequency (male *vs.* female = 47.2% *vs.* 52.8%) in the adenocarcinoma. In contrast, male patients showed a higher frequency of squamous cell carcinoma (male *vs.* female = 89.9% *vs.* 11.1%). As regards cigarette smoking, it was shown that 58% (262/452) had never smoked (never-smokers) and 42% (190/452) were current or former smokers (ever-smokers). Furthermore, never-smoking patients had higher frequency (never-smokers *vs.* ever-smokers = 66.9% *vs.* 33.1%) in the adenocarcinoma and lower frequency (never-smokers *vs.* ever-smokers = 22.2% *vs.* 77.8%) in the squamous cell carcinoma (*p* < 0.001).

The distribution frequency of rs7166348, rs2229765, and rs8038415 of *IGF1R* genotypes in the lung adenocarcinoma and squamous cell carcinoma are shown in Table 2. The alleles with the highest distribution frequency for rs7166348, rs2229765, and rs8038415 of *IGF1R* in recruited patients with NSCLC were heterozygous G/A, homozygous G/G, and heterozygous T/C, respectively. After adjusting for variance, there was no significant difference between the lung adenocarcinoma and squamous cell carcinoma with polymorphisms of the *IGF1R* gene in rs7166348, rs2229765, and rs8038415 when compared with wild-type individuals.

We further investigated the associations between EGFR mutations and patient characteristics. As shown in Table 3, both substitution mutation (L858R) and exon 19 in-frame deletion mutations had higher percentages in female patients (male *vs.* female = 22.8% *vs.* 77.2% and 46.9% *vs.* 53.1%, respectively) and in never-smoker patients (never-smokers *vs.* ever-smokers = 88.6% *vs.* 11.4% and 69.1% *vs.* 30.9%, respectively). The distributions were shown to be significantly different between the control (wild-type) and EGFR mutations for gender (*p* < 0.05) and cigarette smoking status (*p* < 0.05). These results indicate that EGFR mutations are associated with gender and cigarette smoking status.

To clarify the association between the polymorphism of *IFG1R* gene and EGFR mutation in different gender groups, the distribution frequency of *IGF1R* gene (rs7166348, rs2229765, and rs8038415) genotypes of wild-type and EGFR mutation type in lung adenocarcinoma patients was estimated. As shown in Table 4, GA and GA + AA genotypes of *IGF1R* rs2229765 had significant association with EGFR mutation in female lung adenocarcinoma patients (OR = 0.35, 95% CI = 0.15–0.82 and OR = 0.39, 95% CI = 0.17–0.87, respectively). Additionally from Table 4, the polymorphism of *IGF1R* rs2229765 was associated with EGFR mutation only in female lung adenocarcinoma patients. Therefore, further analyses were focused on the association between the polymorphism of *IFG1R* gene and EGFR hotspot mutations in female lung adenocarcinoma patients. Table 5 shows that the GA and GA + AA genotypes of *IGF1R* rs2229765 demonstrated significant association with L858R mutation in female lung adenocarcinoma patients (OR = 0.35, 95% CI = 0.14–0.88 and OR = 0.34, 95% CI = 0.14–0.84, respectively). These results indicate that the polymorphisms of *IGF1R* rs2229765 gene are associated with L858R mutation in female adenocarcinoma patients.

The clinical stage of cancer is a standard way for doctors to understand the time of tumor formation. In this study, the clinicopathological characteristics of lung cancer have been divided into four stages (N0, N1, N2, and N3), dependent on numbers of lymph nodes to which the cancer has spread. We further reveal the association between polymorphisms of *IGF1R* gene and different clinical N stage of lung cancer in different types of patients. As shown in Table 6, GA + AA genotype of *IGF1R* rs7166348 was shown to be significantly associated with the clinical N stage in lung adenocarcinoma (OR = 1.66, 95% CI = 1.07–2.95; *p* = 0.024). Moreover, similar results were found in the wild-type lung adenocarcinoma patients (OR = 2.75, 95% CI = 1.20–6.31; *p* = 0.015). These findings indicate that the polymorphisms of *IGF1R* rs7166348 may be associated with the clinical N stage of lung cancer.

## 3. Discussion

Mutations in the TK domain of the EGFR gene were associated with NSCLC [6,7,8]. Previous studies have reported that EGFR mutations had higher frequency in adenocarcinoma than other types of NSCLCs, in never-smoker as opposed to ever-smokers, and in females rather than males [22,23,24]. Indeed, as shown in Table 3, both substitution mutation (L858R) and in-frame deletion mutation were shown to have a higher frequency in female patients (male *vs.* female = 22.8% *vs.* 77.2% and 46.9% *vs.* 53.1%, respectively) and in never-smoker patients (never-smokers *vs.* ever-smokers = 88.6% *vs.* 11.4% and 69.1% *vs.* 30.9%, respectively). These results were consistent with previous studies indicating that the mutation of EGFR was associated with adenocarcinoma, smoking status, and gender [22,23,24].

The IGF1 signaling pathway mediates multiple cell biological processes including proliferation, differentiation, and metabolism [11]. In previous studies, polymorphism of the *IGF1R* rs2229765 gene was associated with levels of free IGF1 and human longevity [25,26]. In addition, it has been reported that *IGF1R* rs7166348 gene and IGF1 levels are associated with colorectal neoplasia [22]. However, fewer studies were reported for association between *IGF1R* gene polymorphisms and lung cancer. Reinmuth *et al.* [20] have reported the clinical significance of *IGF1R* single nucleotide polymorphisms (SNPs) in resected NSCLC. The results showed that the TT genotype of *IGF1R* 7166348 gave a significantly better survival rate in adenocarcinoma lung cancer. In this study, we have reported the relationship between the polymorphism of the *IGFIR* gene and EGFR mutation in NSCLC. In our studies, three SNPs (rs7166348, rs2229765, and rs8038415) of *IGF1R* were used. The results shown in Table 4 and Table 5 indicated that GA and GA + AA genotypes of the *IGF1R* rs2229765 gene are associated with the L858R mutation of the EGFR in the female lung adenocarcinoma patients. However, the mechanism by which this SNP modulates the roles of female lung adenocarcinoma patients should be further investigated.

Further, we separated the clinical N stages of patients into two subgroup according the lymph nodes metastasis status. As shown in Table 6, GA + AA genotype of *IGF1R* rs7166348 shows significant association with the clinical stage in lung adenocarcinoma (OR = 1.66, 95% CI = 1.07–2.59). Furthermore, GA + AA genotype of *IGF1R* rs7166348 was also shown to be significantly associated with clinical N stage in wild-type lung adenocarcinoma patients (OR = 2.75, 95% CI = 1.20–6.31). These findings implied a relationship linking the polymorphism of the IGFIR gene to the clinical N stage of lung cancer. In addition, previous reports have indicated that rs7166348 in *IGF1R* was more strongly associated with IGF1 levels in colorectal neoplasia patients [22]. It is suggested that rs7166348 SNP may increase the activity or expression of IGF1 at the NSCLC progressive stage. However, the underlying mechanism should be elucidated in the future.

## 4. Materials and Methods

### 4.1. Patient Specimens

For the 2012–2015 period, we recruited 452 patients with NSCLC, including 362 adenocarcinoma lung cancer and 90 squamous cell carcinoma lung cancer patients, at Cheng-Ching General Hospital in Taichung, Taiwan. Medical information of the patients, including Tumor, Node, Metastasis (TNM) clinical staging, primary tumor size, lymph node involvement, and histologic grade, was obtained from their medical records. This study was approved by the Institutional Review Board of Cheng-Ching General Hospital (No. HP120009; 22 September 2012), and informed consent was obtained from all subjects.

### 4.2. Genomic DNA Extraction and Insulin-Like Growth Factor 1 Receptor (IGF1R) Genotyping

Venous blood from each subject was drawn into Vacutainer tubes containing EDTA and stored at 4 °C. Genomic DNA was extracted by QIAamp DNA blood mini kits (Qiagen, Valencia, CA, USA) according to the manufacturer’s instructions. Allelic discrimination of IGF1R rs7166348, rs2229765, and rs8038415 gene polymorphism was assessed with the ABI StepOne™ Real-Time PCR System (Applied Biosystems, Foster City, CA, USA) and analyzed using SDS vers. 3.0 software (Applied Biosystems) with the TaqMan assay [21].

### 4.3. Statistical Analysis

The distributions of demographic characteristics and genotype frequencies between adenocarcinoma lung cancer and squamous cell carcinoma lung cancer, as well as clinicopathological features in different genotypes, were analyzed with a χ^2^-test. The odds ratio and 95% CIs of the association between the genotype frequencies and EGFR mutation risk and the clinical pathological characteristics were estimated using multiple logistic regression models after controlling for other covariates. A *p*-value of <0.05 was considered statistically significant. The data were analyzed with SAS statistical software (SAS Institute Inc., Cary, NC, USA).

## 5. Conclusions

In conclusion, the mutation of EGFR was associated with adenocarcinoma, smoking status, and gender and the polymorphisms of the *IGF1R* rs2229765 gene were associated with the L858R mutation of EGFR in female lung adenocarcinoma patients who had never smoked. Furthermore, the polymorphism of *IGFIR* rs7166348 gene was associated with the clinical N stage of lung cancer. However, we are still lacking a mechanistic explanation for this phenomenon, which should be further investigated in the future.

## Figures and Tables

**Table 1:** Demographics and clinical characteristics of 452 patients with lung adenocarcinoma and lung squamous cell carcinoma.

Variable	All Cases (*N* = 452) *n* (%)	Adenocarcinoma (*N* = 362) *n* (%)	Squamous Cell Carcinoma (*N* = 90) *n* (%)	*p*-Value
**Age**				
<30	3 (0.7%)	3 (0.8%)	0 (0%)	*p* = 0.732
30–39	9 (2.0%)	8 (2.2%)	1 (1.1%)	
40–49	40 (8.8%)	33 (9.1%)	7 (7.8%)	
50–59	96 (21.2%)	80 (22.1%)	16 (17.8%)	
60–69	99 (21.9%)	76 (21.0%)	23 (25.6%)	
≥70	205 (45.4%)	162 (44.8%)	43 (47.8%)	
Mean ± SD	66.27 ± 13.85	65.82 ± 13.95	68.06 ± 13.37	*p* = 0.171
**Gender**				
Male	251 (55.5%)	171 (47.2%)	80 (88.9%)	*p* < 0.001
Female	201 (44.5%)	191 (52.8%)	10 (11.1%)	
**Cigarette Smoking Status**				
Never-smoker	262 (58.0%)	242 (66.9%)	20 (22.2%)	*p* < 0.001
Ever-smoker	190 (42.0%)	120 (33.1%)	70 (77.8%)	
pack-years	39.22 ± 29.62	33.29 ± 28.80	52.95 ± 26.98	*p* < 0.001
**Disease Stage**				
IA	45 (10.0%)	41 (11.3%)	4 (4.4%)	*p* < 0.001
IB	57 (12.6%)	52 (14.4%)	5 (5.6%)	
IIA	27 (6.0%)	21 (5.8%)	6 (6.7%)	
IIB	10 (2.2%)	7 (1.9%)	3 (3.3%)	
IIIA	40 (8.8%)	29 (8.0%)	11 (12.2%)	
IIIB	67 (14.8%)	39 (10.8%)	28 (31.1%)	
IV	206 (45.6%)	173 (47.8%)	33 (36.7%)	
**Cell Differentiation**				
Good	42 (9.3%)	39 (10.8%)	3 (3.3%)	*p* < 0.001
Moderate	326 (72.1%)	280 (77.3%)	46 (51.1%)	
Poor	84 (18.6%)	43 (11.9%)	41 (45.6%)	

Mean ± SD: Mean ± standard deviation.

**Table 2:** Distribution frequency of insulin-like growth factor 1 receptor (IGF1R) genotypes in 362 lung adenocarcinoma and 90 lung squamous cell carcinoma.

Variable	Adenocarcinoma (*N* = 362) (%)	Squamous Cell Carcinoma (*N* = 90) (%)	Odds Ratio (95% Confidence Interval)	Adjusted Odds Ratio (95% CI)
***IGF1R* rs7166348**				
GG	117 (32.3%)	34 (37.8%)	1.00	1.00
GA	189 (52.2%)	44 (48.9%)	0.80 (0.48–1.33)	0.67 (0.39–1.18)
AA	56 (15.5%)	12 (13.3%)	0.74 (0.36–1.53)	0.69 (0.31–1.54)
GA + AA	245 (67.7%)	56 (62.2%)	0.79 (0.49–1.27)	0.68 (0.40–1.15)
***IGF1R* rs2229765**				
GG	168 (46.4%)	43 (47.8%)	1.00	1.00
GA	155 (42.8%)	34 (37.8%)	0.86 (0.52–1.41)	0.90 (0.52–1.55)
AA	39 (10.8%)	13 (14.4%)	1.30 (0.64–2.65)	2.08 (0.92–4.72)
GA + AA	194 (53.6%)	47 (52.2%)	0.95 (0.60–1.50)	1.08 (0.65–1.79)
***IGF1R* rs8038415**				
TT	90 (24.9%)	27 (30.0%)	1.00	1.00
TC	186 (51.4%)	37 (41.1%)	0.66 (0.38–1.16)	0.61 (0.33–1.13)
CC	86 (23.7%)	26 (28.9%)	1.01 (0.55–1.86)	1.02 (0.52–2.03)
TC + CC	272 (75.1%)	63 (70.0%)	0.77 (0.46–1.29)	0.73 (0.42–1.29)

**Table 3:** Demographics and clinical characteristics of 279 patients in lung adenocarcinoma with epidermal growth factor receptor (EGFR) mutation status.

Variable	Wild Type (*N* = 110) *n* (%)	L858R (*N* = 79) *n* (%)	In-Frame Deletion (*N* = 81) *n* (%)	Others (*N* = 9) *n* (%)
**Age**				
<30	1 (0.9%)	0 (0%)	1 (1.2%)	0 (0%)
30–39	3 (2.7%)	0 (0%)	2 (2.5%)	0 (0%)
40–49	11 (9.9%)	6 (7.6%)	10 (12.3%)	0 (0%)
50–59	21 (18.9%)	16 (20.3%)	27 (33.3%)	1 (11.1%)
60–69	26 (23.4%)	16 (20.3%)	14 (17.3%)	1 (11.1%)
≥70	49 (44.1%)	41 (51.9%)	27 (33.3%)	7 (77.8%)
Mean ± SD	65.36 ± 13.42	68.18 ± 12.56	62.31 ± 14.01 ^b^	75.56 ± 9.04
**Gender**				
Male	67 (60.4%)	18 (22.8%) ^a^	38 (46.9%) ^b^	4 (44.4%)
Female	44 (39.6%)	61 (77.2%)	43 (53.1%)	5 (55.6%)
**Cigarette Smoking Status**				
Never-smoker	50 (45.0%)	70 (88.6%) ^a^	56 (69.1%) ^a^	5 (55.6%)
Ever-smoker	61 (55.0%)	9 (11.4%)	25 (30.9%)	4 (44.4%)
PPK	46.32 ± 28.21	12.76 ± 20.81 ^a^	21.15 ± 23.15 ^a^	46.00 ± 28.15
**Disease Stage**				
IA	11 (9.9%)	6 (7.6%)	11 (13.6%)	0 (0%)
IB	9 (8.1%)	13 (16.5%)	9 (11.1%)	1 (11.1%)
IIA	5 (4.5%)	4 (5.1%)	3 (3.7%)	0 (0%)
IIB	1 (0.9%)	0 (0%)	0 (0%)	0 (0%)
IIIA	10 (9.0%)	7 (8.9%)	4 (4.9%)	0 (0%)
IIIB	17 (15.3%)	10 (12.7%)	7 (8.6%)	2 (22.2%)
IV	58 (52.3%)	39 (49.4%)	47 (58.0%)	6 (66.7%)
**Cell Differentiation**				
Good	8 (7.2%)	11 (13.9%) ^a^	8 (9.9%) ^a^	2 (22.2%)
Moderate	80 (72.1%)	63 (79.7%)	68 (84.0%)	7 (77.8%)
Poor	23 (20.7%)	5 (6.3%)	5 (6.1%)	0 (0%)

^a^ Significant difference when compared with wild-type group, *p*-value < 0.05; ^b^ Significant difference when compared with L858R group, *p*-value < 0.05.

**Table 4:** Distribution frequency of IGF1R genotypes, the 111 EGFR wild type, and the 169 EGFR mutation type in lung adenocarcinoma patients.

Variable	All Cases (*N* = 280)		
	Wild Type (*N* = 111) (%)	Mutation Type (*N* = 169) (%)	AOR (95% CI)
***IGF1R* rs7166348**			
GG	36 (32.4%)	50 (29.6%)	1.00
GA	59 (53.2%)	89 (52.7%)	1.30 (0.72–2.32)
AA	16 (14.4%)	30 (17.7%)	1.49 (0.67–3.28)
GA + AA	75 (67.6%)	119 (70.4%)	1.34 (0.77–2.34)
***IGF1R* rs2229765**			
GG	48 (43.2%)	81 (47.9%)	1.00
GA	50 (45.0%)	69 (40.8%)	0.75 (0.43–1.29)
AA	13 (11.8%)	19 (11.3%)	0.69 (0.29–1.65)
GA + AA	63 (56.8%)	88 (52.1%)	0.73 (0.44–1.24)
***IGF1R* rs8038415**			
TT	29 (26.1%)	42 (24.9%)	1.00
TC	51 (45.9%)	88 (52.1%)	1.23 (0.66–2.30)
CC	31 (27.9%)	39 (23.0%)	1.11 (0.54–2.27)
TC + CC	80 (73.9%)	127 (75.1%)	1.19 (0.66–2.13)
**Male (*N* = 127)**			
***IGF1R* rs7166348**			
GG	21 (31.3%)	14 (23.3%)	1.00
GA	38 (56.7%)	34 (56.7%)	2.08 (0.63–6.95)
AA	8 (12.0%)	12 (20.0%)	4.70 (0.90–24.52)
GA + AA	46 (68.7%)	46 (76.7%)	2.48 (0.99–6.20)
***IGF1R* rs2229765**			
GG	35 (43.2%)	28 (46.7%)	1.00
GA	26 (45.0%)	26 (43.3%)	1.27 (0.57–2.82)
AA	6 (11.8%)	6 (10.0%)	1.02 (0.25–4.17)
GA + AA	32 (47.8%)	32 (53.3%)	1.22 (0.57–2.59)
***IGF1R* rs8038415**			
TT	16 (23.9%)	16 (26.7%)	1.00
TC	34 (50.7%)	27 (45.0%)	0.93 (0.36–2.40)
CC	17 (25.4%)	17 (28.3%)	1.24 (0.44–3.56)
TC + CC	51 (76.1%)	44 (73.3%)	1.04 (0.43–2.51)
**Female (*N* = 153)**			
***IGF1R* rs7166348**	15 (34.1%)	36 (33.0%)	1.00
GG	21 (47.7%)	55 (50.5%)	1.21 (0.53–2.77)
GA	8 (18.2%)	18 (16.5%)	1.14 (0.37–3.49)
AA	29 (65.9%)	73(67.0%)	1.19 (0.54–2.60)
GA + AA			
***IGF1R* rs2229765**	13 (29.5%)	53 (48.6%)	1.00
GG	24 (54.5%)	43 (39.4%)	0.35 (0.15–0.82)
GA	7 (16.0%)	13 (12.0%)	0.54 (0.16–2.85)
AA	31 (70.5%)	56 (51.4%)	0.39 (0.17–0.87)
GA + AA			
***IGF1R* rs8038415**			
TT	13 (29.5%)	26 (23.9%)	1.00
TC	17 (38.6%)	61 (55.9%)	1.61 (0.66–3.97)
CC	14 (31.9%)	22 (20.2%)	0.84 (0.30–2.35)
TC + CC	31 (70.5%)	83 (76.1%)	1.30 (0.56–2.98)

**Table 5:** The associations between the polymorphisms of *IGF1R* and the EGFR hotspot mutations in female lung adenocarcinoma patients.

Variable	Wild Type	L858R		Exon 19 in-Frame Deletion	
	(*N* = 44) *n* (%)	(*N* = 61) (%)	AOR (95% CI)	(*N* = 43) (%)	AOR (95% CI)
***IGF1R* rs7166348**					
GG	15 (34.1%)	22 (34.1%)	1.00	12 (27.9%)	1.00
GA	21 (47.7%)	29 (47.7%)	1.17 (0.45–3.01) *p* = 0.746	23 (53.5%)	1.47 (0.53–4.04) *p* = 0.458
AA	8 (18.2%)	10 (18.2%)	1.12 (0.31–4.11) *p* = 0.864	8 (18.6%)	1.38 (0.36–5.38) *p* = 0.640
GA + AA	29 (65.9%)	39 (63.9%)	1.16 (0.47–2.86) *p* = 0.751	31 (72.1%)	1.45 (0.55–3.77) *p* = 0.452
***IGF1R* rs2229765**					
GG	13 (29.5%)	34 (55.7%)	1.00	15 (34.9%)	1.00
GA	24 (54.5%)	22 (36.1%)	0.35 (0.14–0.88) *p* = 0.026	21 (48.8%)	0.53 (0.19–1.49) *p* = 0.227
AA	7 (16.0%)	5 (8.2%)	0.35 (0.08–1.52) *p* = 0.160	7 (16.3%)	0.85 (0.20–3.66) *p* = 0.829
GA + AA	31 (70.5%)	27 (44.3%)	0.34 (0.14–0.84) *p* = 0.020	28 (65.1%)	0.58 (0.22–1.58) *p* = 0.290
***IGF1R* rs8038415**					
TT	13 (29.5%)	15 (24.6%)	1.00	10 (23.3%)	1.00
TC	17 (38.6%)	33 (54.1%)	1.77 (0.65–4.85) *p* = 0.268	25 (58.1%)	1.47 (0.50–4.35) *p* = 0.484
CC	14 (31.9%)	13 (21.3%)	1.00 (0.32–3.16) *p* = 0.999	8 (18.6%)	0.68 (0.18–2.53) *p* = 0.560
TC + CC	31 (70.5%)	46 (75.4%)	1.45 (0.57–3.66) *p* = 0.434	33 (76.7%)	1.16 (0.42–3.21) *p* = 0.780

**Table 6:** Associations between polymorphic genotypes of *IGF1R* rs7166348 and clinicopathologic characteristics of lung cancer.

Variable Genotypefrequencies	Clinical Stage			
	N0 + N1	N2 + N3	OR (95% CI)	*p*-Value
**All Cases (*N* = 452)**	**(*N* = 151)**	**(*N* = 185)**		
*IGF1R* rs7166348 GG	68 (45.0%)	116 (38.5%)	1.00	
GA + AA	83 (55.0%)	185 (61.5%)	1.31 (0.88–1.94)	*p* = 0.185
**adenocarcinoma (*N* = 362)**	**(*N* = 117)**	**(*N* = 245)**		
*IGF1R* rs7166348 GG	62 (53.0%)	99 (40.4%)	1.00	
GA + AA	55 (47.0%)	146 (59.6%)	1.66 (1.07–2.59)	*p* = 0.024
**Squamous Cell Carcinoma (*N* = 90)**	**(*N* = 34)**	**(*N* = 56)**		
*IGF1R* rs7166348 GG	6 (17.6%)	17 (30.4%)	1.00	
GA + AA	28 (82.4%)	39 (69.6%)	0.49 (0.17–1.40)	*p* = 0.180
**Wild Type (*N* = 111)**	**(*N* = 36)**	**(*N* = 75)**		
*IGF1R* rs7166348 GG	18 (50.0%)	20 (26.7%)	1.00	
GA + AA	18 (50.0%)	55 (73.3%)	2.75 (1.20–6.31)	*p* = 0.015
**L858R (*N* = 79)**	**(*N* = 28)**	**(*N* = 51)**		
*IGF1R* rs7166348 GG	13 (46.4%)	21 (41.2%)	1.00	
GA + AA	15 (53.6%)	30 (58.8%)	1.24 (0.49–3.13)	*p* = 0.652
**In-Frame Deletion (*N* = 81)**	**(*N* = 20)**	**(*N* = 61)**		
*IGF1R* rs7166348 GG	5 (25.0%)	21 (34.4%)	1.00	
GA + AA	15 (75.0%)	40 (65.6%)	0.64 (0.20–1.99)	*p* = 0.433

## Abbreviations

IGF1	insulin-like growth factor 1
IGF1R	insulin-like growth factor 1 receptor
EGFR	epidermal growth factor receptor
NSCLC	non-small-cell lung carcinoma
